# Perpendicular probing and screwing technique: A simple method for accurate pedicle screw placement based on the human internal reference frame for angle estimation

**DOI:** 10.1371/journal.pone.0277229

**Published:** 2022-11-28

**Authors:** Go Kato, Satoshi Baba, Kenichi Kawaguchi, Takeshi Watanabe, Takao Mae, Shinji Tomari

**Affiliations:** 1 Department of Orthopedic Surgery, Japanese Red Cross Fukuoka Hospital, Fukuoka, Japan; 2 Department of Spine Surgery, Saga Medical Center, Koseikan, Saga, Japan; 3 Department of Orthopedic Surgery, Kyushu University Graduate School of Medical Sciences, Fukuoka, Japan; 4 Department of Orthopedic Surgery, Watanabe Orthopedic Hospital, Fukuoka, Japan; 5 Department of Orthopedic Surgery, Saga Medical Center, Koseikan, Saga, Japan; University of Vigo, SPAIN

## Abstract

The pedicle screw (PS) is widely used for spinal fixation surgery. However, PS malpositioning can cause critical complications; thus, the accuracy of ascertaining PS trajectory is paramount. This study aimed to demonstrate the accuracy and safety of a simple and cost-effective PS placement technique using a human internal reference frame for angle estimation. *Ex vivo* lumbar porcine spine samples were fixed to a wooden board with rostrocaudal and mediolateral rotational angles adjusted by two angle vises. PS entry points (EPs) were identified using clear anatomical vertebral landmarks. PS placement was performed on one side using the perpendicular probing and screwing technique (PPST), wherein the attitude angle of the sample was adjusted such that the longitudinal axis of the target pedicle was perpendicular to the ground. The pedicle probe and PS driver were manually maintained perpendicular to the ground during probing and PS placement. PS placement on the contralateral side was performed freehand as a control. Offsets between the preoperatively planned and implanted PS rotational angles measured using computed tomography for PPST and freehand method were analyzed. Pedicle wall penetration was also evaluated. The mean ± standard error of the medial rotational offsets was 5.83° ± 0.57° in the freehand group versus 2.89° ± 0.31° in the PPST group (p <0.001), and the rostrocaudal rotational offsets were 4.81° ± 0.65° in the freehand group versus 2.92° ± 0.45° in the PPST group (p = 0.01). The mean pedicle wall penetration distance was significantly reduced by PPST (0.28 ± 0.12 mm *vs* 0.80 ± 0.17 mm in the freehand group, p = 0.0071). Thus, PPST improved PS positioning accuracy, resulting in reduced pedicle wall penetration and increased PS placement safety. This simple technique is also potentially cost-effective for institutions without computer-assisted surgical systems.

## Introduction

Pedicle screw (PS) mispositioning in spinal fusion surgery can cause injury to the spinal cord, nerve root, or vasculature [1, 2]. Therefore, increasing the accuracy and safety of PS placement is an important issue in this field. For safe and accurate PS placement, both the entry point (EP) coordinates and PS trajectory, regulated by the rostrocaudal and mediolateral rotational angles against the vertebral axes, are critical. When using the conventional free-hand technique for PS insertion, CT images of the target vertebrae are obtained preoperatively, the rostrocaudal and mediolateral rotational angles of the central axis of the isthmus of the pedicles of the vertebrae against the ground level are measured, and an entry point for PS is identified as the intersection of the posterior bone element surface and the central axis of isthmus of the pedicle. During the PS insertion procedure, the operator tries to reproduce the preoperatively planned ideal location of the entry point and the rostrocaudal and mediolateral rotational angles of the axis of the pedicle probe and PS without the jig. A PS with relatively large diameter is selected to increase pull-out strength and avoid implant failure. Under these conditions, pedicle wall penetration occurs frequently, especially at the cervical, middle thoracic, and upper lumbar vertebrae, in which the diameter and length of the narrowest portion of the pedicle are relatively small and long, respectively.

A recent analysis with a relatively large sample size demonstrated that the overall correct positioning rate for the freehand group of thoracic and lumbo-sacral PS was 72.6% that was significantly lower than intraoperative CT-assisted navigation group (96.5%) [3].

However, the detailed conditions in which clinically unacceptable PS malpositioning occurs by the freehand technique are yet to be revealed. Therefore, the accuracy and safety of PS placement can presumably be increased under modified and selected conditions, in which the PS trajectory made freehand is practically reliable. To obtain accurate PS trajectories using the conventional freehand technique, the axes of the pedicle probe and screwdrivers should correspond to the preoperatively planned trajectory by intuitively rotating these tools in the rostral, caudal, medial, or lateral directions.

Regarding the rotational orientation perception of lines, previous papers [4–6] demonstrated that the effect of line orientation sensitivity on a just-noticeable difference in the angle was the highest at 90° (vertical line) and 0° (horizontal line), and the lowest at 45° to the ground. Xu et al. [4] hypothesized that the human visual system uses an orthogonal internal reference frame in angle perception acquired by the postnatal visual experience and concluded that the combination of the internal reference frame. Weber’s law in angle perception reasonably explained the phenomenon, namely, when a given line is near perpendicular, the observer can almost certainly notice the change between the orientations of the given line orientation and perpendicular axis, allowing the observer to distinguish whether the line is perpendicular. However, when the given line is gradually inclined, quantifying the angle of inclination becomes more difficult, and the angular offset between the angle of the line and the angle intuitively estimated by the observer increases [7].

**Fig 1:**
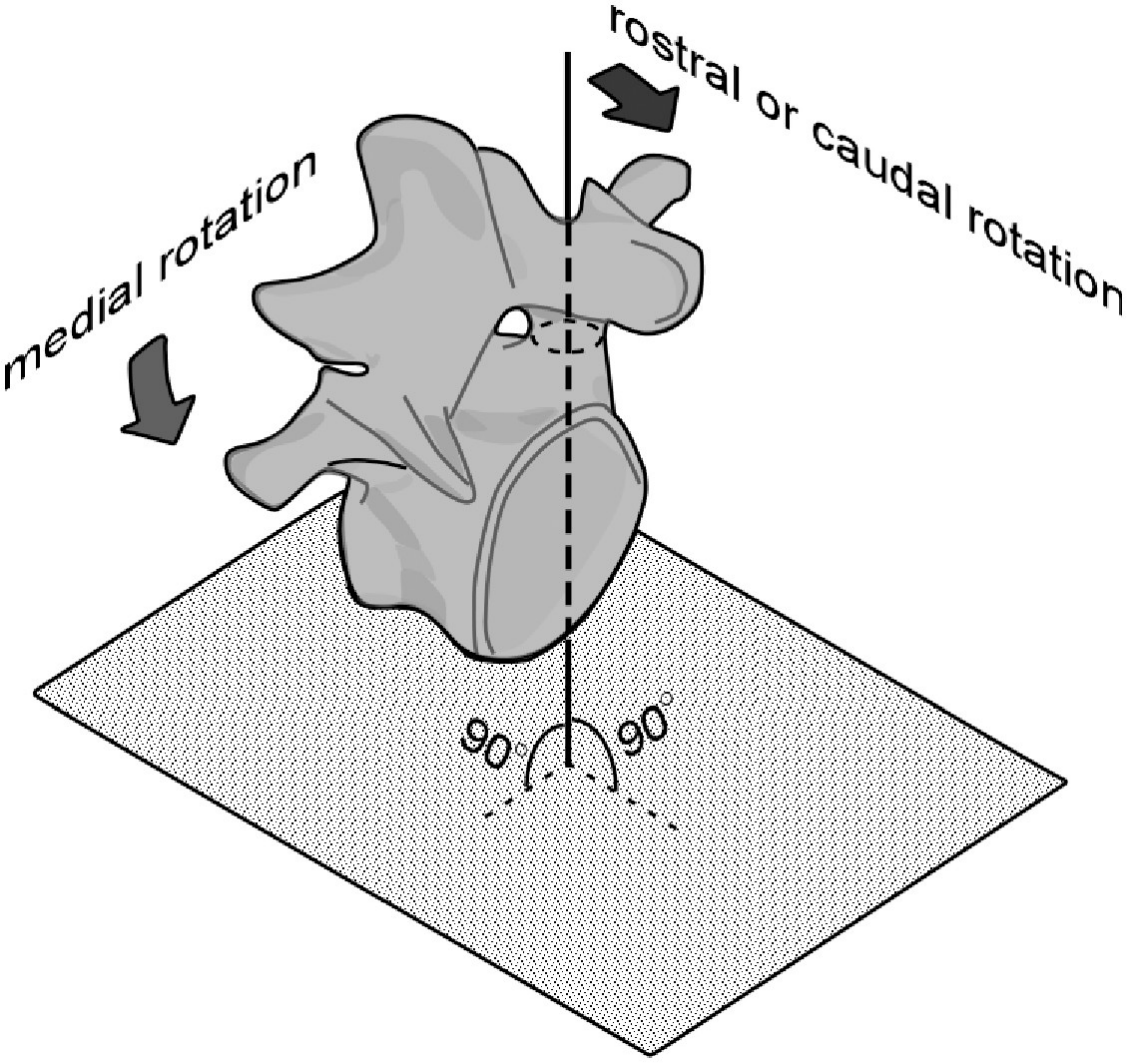
Schematic diagram of positioning the longitudinal axis of the target pedicle perpendicular to the ground by medially and rostrally or caudally rotating the spine.

In this study, we aimed to test the safety and accuracy of the PS placement method by applying the orthogonal internal reference frame concept in angle perception by performing simulated PS placement surgery in porcine lumbar spine samples. Specifically, we investigated whether the offset between the preoperatively planned and postoperatively measured implanted PS trajectories could be recovered if the axis of the target pedicle was placed perpendicular to the ground by caudal, rostral, lateral, or medial rotations of the sample (Fig 1), resulting in reduced pedicle wall penetration during PS placement.

## Materials and methods

The study protocol was reviewed and approved by the research ethics committee of Saga Medical Center, Koseikan. Five *ex vivo* specific-pathogen-free specimens, including L1–L6 lumbar spines and paraspinal muscles, harvested from 10-week-old female pig cadavers, were obtained from the Intervention Technical Center (Kobe, Japan). The methods used in this study were performed according to the protocols described in our previous study [7].

### PS insertion in a porcine lumbar spine

The methods used for the PS insertion experiments were similar to those previously described [7]. Briefly, the lumbar spine before and after PS insertion surgery was scanned using a 64-row multi-detector CT unit and viewed in the multiplanar reconstruction mode (MPR) on a ZioCube digital imaging and communications in medicine (DICOM) viewer (Ziosoft Co. Ltd., Tokyo, Japan). PSs extracted from the patients were reused. The largest but ≥1 mm smaller-size screws were selected. Screw length was not considered because the focus was on screw placement accuracy at the pedicle.

### Preoperative planning

All the screw trajectories were planned by the same spine surgeon (GK). The ideal trajectory of the PS placement was defined as a line parallel to the axis of the narrowest portion of the pedicle on an arbitrary axial plane (AAP) parallel to the cranial endplate (CE) and passing through the middle of the rostral and caudal ends of the narrowest portion of the pedicle. The EP was defined as the point at which the ideal trajectory and rostral articular process base crossed.

The rostral tilting of the AAP parallel to the CE on which the ideal trajectory was defined and the wooden board surface on which the cadaver was mounted was measured on a DICOM viewer and was defined as alpha (α). The angle of medial tilting of the ideal trajectory on the AAP parallel to the CE was measured and defined as beta (β). The EP coordinates, the lateral tip of the transverse process (TTP), and the rostral tip of the rostral articular process (TRAP) were measured using a DICOM viewer. The distances from TTP to EP and from TRAP to EP were calculated.

### Procedure for freehand PS placement and the perpendicular probing and screwing technique

**Fig 2:**
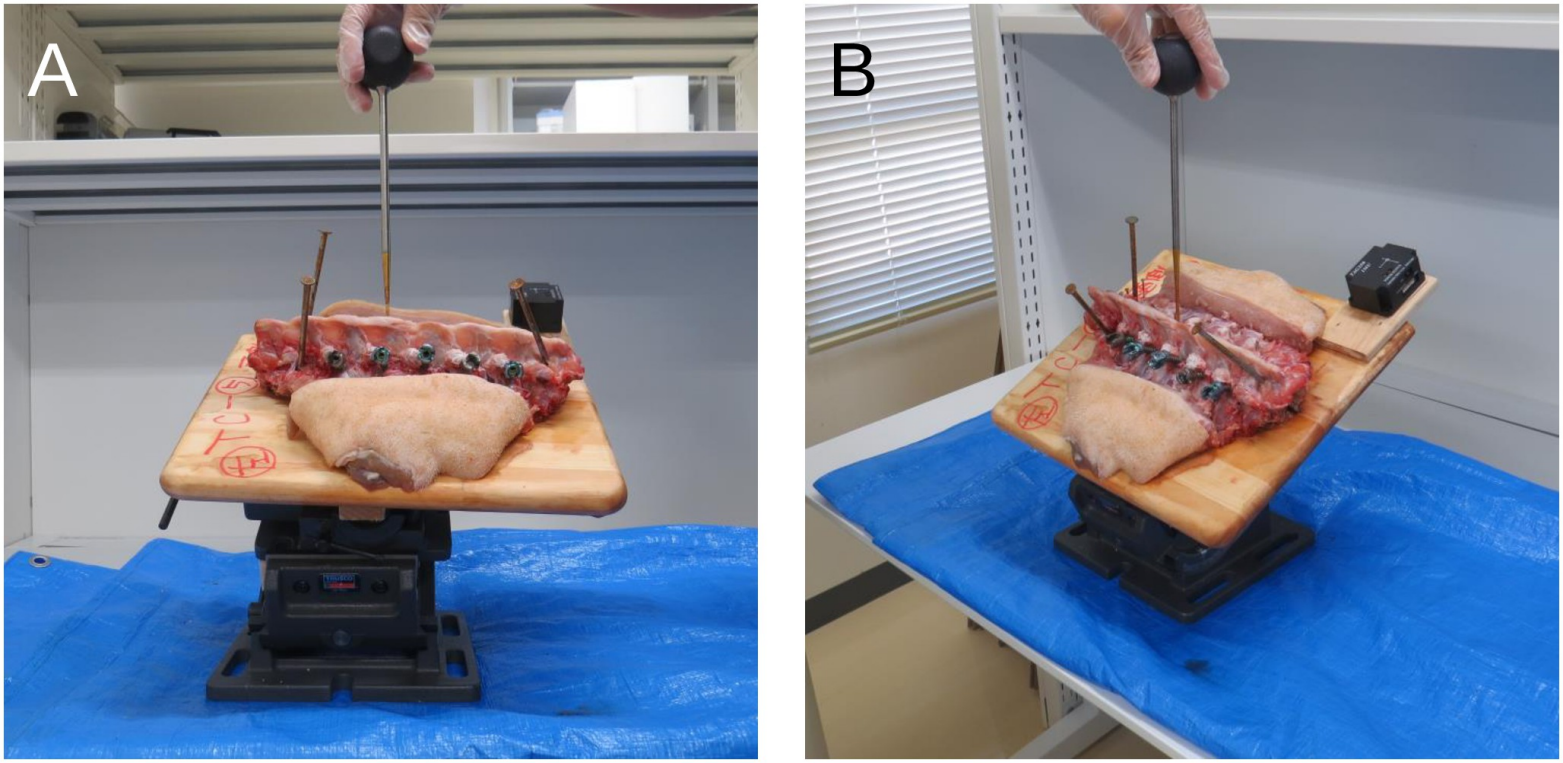
Photographs from a lateral view (A) and from diagonally above (B), the longitudinal axis of the target pedicle perpendicularly positioned to the ground by medially and rostrally rotating the sample by adjusting the two angle vise clamps attached to the bottom of the board on which the cadaver was mounted. The upper angle vise clamp was fastened by the lower angle vise clamp, and the rotating planes were orthogonal to each other.

In one sample, PSs were placed freehand on one side and using the perpendicular probing and screwing technique (PPST) on the contralateral side by one of the authors (S.B., 1 year of experience in spine surgery). A compass was used to find an EP by measuring the intersection of the arcs with the radii of the distances from TTP to EP and from TRAP to EP. In the PPST, we sought to ensure that the ideal PS trajectory was perpendicular to the ground by (1) caudally rotating the board at an angle of α by tilting an upper angle vise clamping the board, and then (2) laterally rotating the upper angle vise and the board at an angle of β by tilting a lower angle vise clamping the upper angle vise (Fig 2A and 2B). To avoid micro-rotations of the spine caused by applying loads during pedicle probing or PS insertion, surgeons using PPST should confirm whether the axes of the pedicle probe or screw were perpendicular to the ground when these loads were released.

### Postoperative evaluation

**Fig 3:**
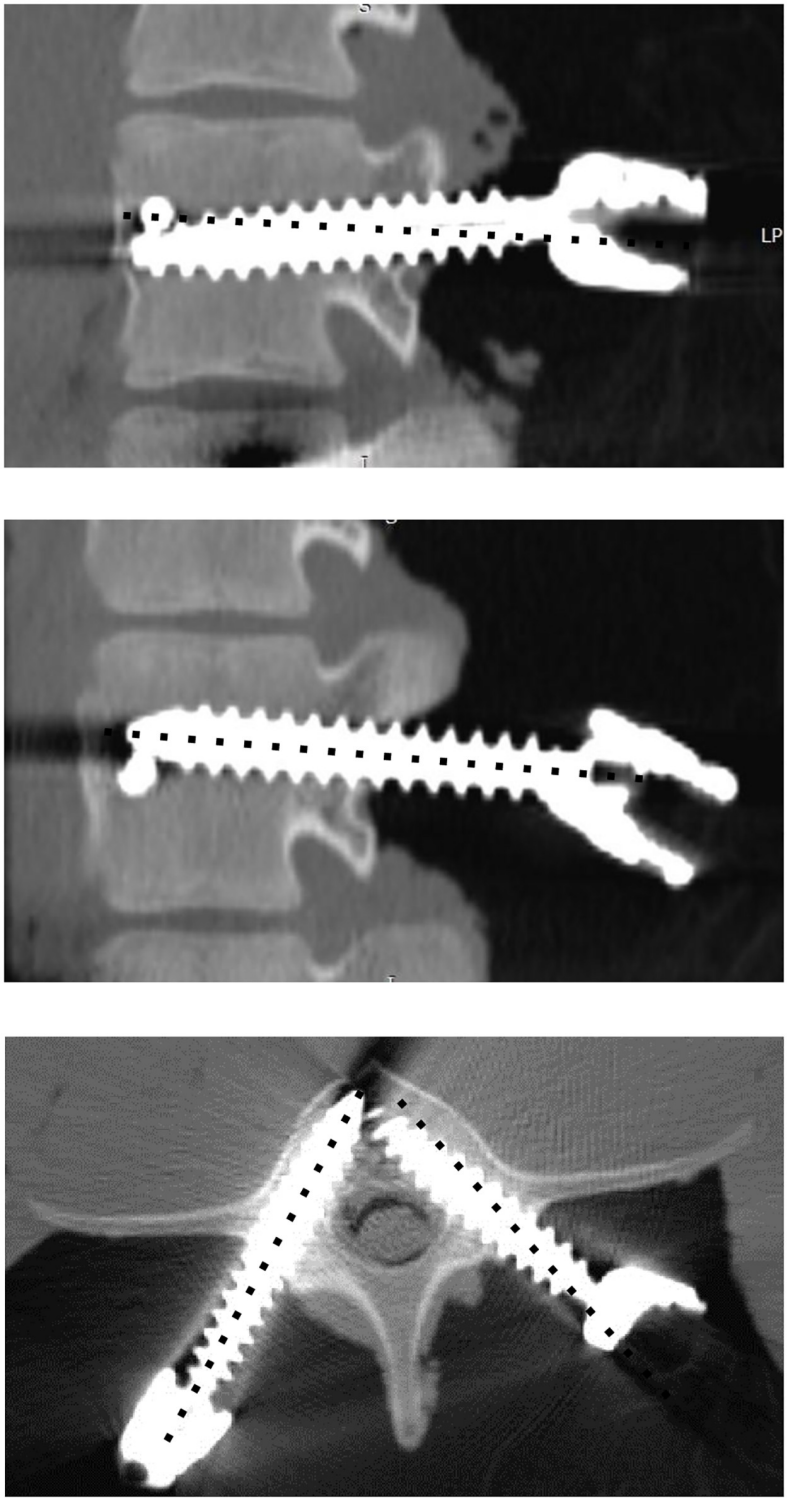
Parasagittal (upper and middle) and arbitrary axial planes (lower) obtained by postoperative computed tomography scans. The black dotted lines indicate the planned pedicle screw (PS) trajectories. The upper panel shows that the rostrocaudal angle of PS placed freehand caudally deviated relative to the planned angle, whereas the middle panel shows that the rostrocaudal angle of PS placed by perpendicular probing and screwing technique (PPST) was almost parallel to the planned angle. The lower panel shows that the mediolateral angle of the right PS placed by PPST was almost parallel to the planned angle, whereas the mediolateral angle of the left PS placed freehand medially deviated to the planned angle.

A postoperative CT scan was performed, and the rostral and medial rotational angles of the implanted PSs were measured in MPR mode using a DICOM viewer (Fig 3). The preoperative and postoperative angles were compared. The screw trajectory was checked for cortical bone breaches and the protrusion distance of the PS from the pedicle cortical bone was evaluated. We evaluated the offsets between the inserted screw EP and planned EP in the freehand and PPST groups by comparing the pre- and postoperative EP–TRAP and EP–TTP distances.

### Sample sizes and statistical evaluation

The results are expressed as means ± standard error. A one-tailed t-test with α = 0.05, power = 0.08, and effect size from medium (0.5) to large (0.8) [8] required 21–51 samples for statistical significance (R version; 3.4.4 package pwr); thus, we aimed to obtain data from that range of samples.

Screw placement was not performed when the nails for fixing the samples to a board were observed on the ideal trajectory during preoperative planning. Data were also excluded when the drilled EPs were observed to be >2 mm away from the planned EPs in the postoperative analyses of EP coordinates. Consequently, 54 screws were implanted into 9 lumbar spine samples (L1–L6), 27 by freehand, and 27 using PPST.

The freehand and PPST-assisted results were compared using an independent two-sample t-test performed using Origin 8.6 (Origin Lab Corporation, Northampton, MA, USA). The significance level was set at p = 0.05.

## Results

### Improved reproducibility of the preoperatively planned PS trajectory by PPST

No significant differences in the average planned rostrocaudal and medial rotation angles (Table 1), and no differences between the planned and postoperative distances between EP and TRAP or TTP (Table 2), average PS diameters, narrowest pedicle width, and the PS size to the narrowest pedicle width ratio (Table 3) were observed between the freehand and PPST groups. The results further suggested no significant difference between the freehand and PPST groups in the parameters of the ideal trajectories for PS, the coordinate errors of EP, and the space available for the PS at the narrowest portion of the pedicle.

**Table 1:** Average planned rotational angles of the freehand and PPST groups.

Planned rotation	Freehand n = 27	PPST n = 27	*p*-value
**Rostral or caudal (°)**	−0.92 ± 0.88	0.79 ± 1.12	0.23
**Medial (°)**	38.1 ± 1.34	37.3 ± 1.21	0.17

PPST, perpendicular probing and screwing technique. Data are expressed as means ± standard error.

**Table 2:** Average differences between the pre- and postoperative distances between EP and TRAP or TTP.

Position displacement (distance)	Freehand n = 27	PPST n = 27	*p*-value
**EP–TRAP (mm)**	1.15 ± 0.25	1.55 ± 0.22	0.22
**EP–TTP (mm)**	2.27 ± 0.25	2.33 ± 0.29	0.88

EP, endpoint; TRAP, tip of the rostral articular process; TTP, transverse process; PPST, perpendicular probing and screwing technique. Data are expressed as means ± standard error.

**Table 3:** Average diameters of the PS, the narrowest width of the pedicle, and the ratio of the PS size to the narrowest pedicle width.

Sizes and ratio	Freehand n = 27	PPST n = 27	*p*-value
**PS diameter (mm)**	5.91 ± 0.11	5.89 ± 0.12	0.80
**pedicle width (mm)**	7.06 ± 0.11	7.05 ± 0.13	0.96
**Ratio (%)**	83.6 ± 0.49	83.5 ± 0.73	0.88

PS, pedicle screw; PPST, perpendicular probing and screwing technique. Data are expressed as means ± standard error.

**Fig 4:**
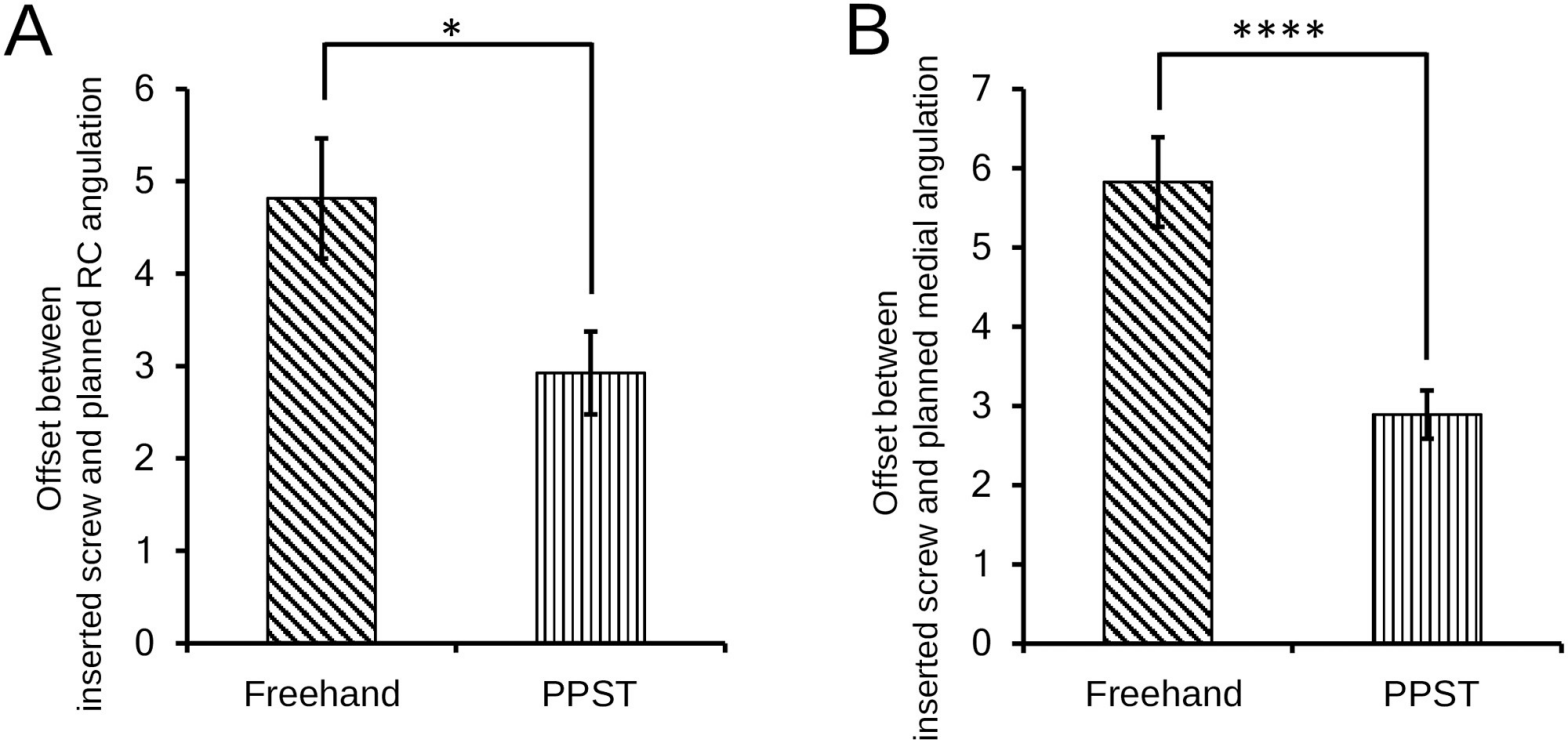
(A) Mean offsets between the inserted pedicle screw (PS) and preoperatively planned rostrocaudal rotations in the freehand and perpendicular probing and screwing technique (PPST) groups. (B) Mean offsets between the inserted PS and preoperatively planned mediolateral rotations in the freehand and PPST groups. *p <0.05; ****p <0.0001.

The mean offset between the preoperative PS placement and postoperative implanted PS rostrocaudal rotations was 4.81° ± 0.65° in the freehand group and 2.92° ± 0.45° in the PPST group (p = 0.0116) (Fig 4A), indicating that PPST could significantly reduce PS rostrocaudal rotational deviation. Furthermore, the mean offset between the preoperatively planned and postoperatively measured medial rotations on the perpendicular PS axis was 5.83° ± 0.57° in the freehand group and 2.89 ± 0.31° in the PPST group, indicating that the planned PS medial rotation was reproduced significantly better by PPST than by freehand (p <0.0001) (Fig 4B).

### PPST significantly reduced the pedicle cortical breach distance

**Fig 5:**
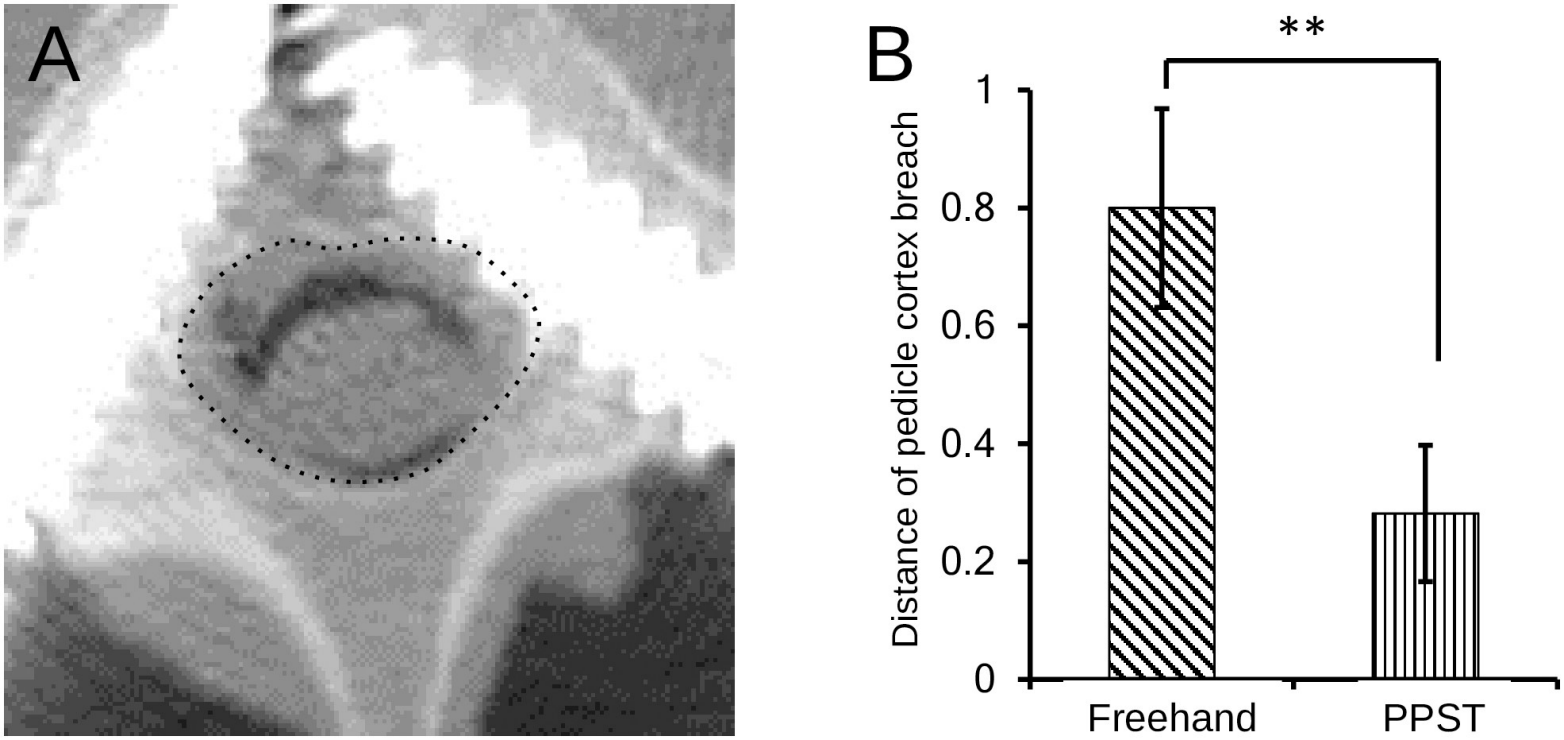
(A) An enlarged image of the lower panel of Fig 3, demonstrating that the left pedicle screw (PS) placed by perpendicular probing and screwing technique (PPST) is contained in both medial and lateral cortical layers of a pedicle, whereas the right PS placed by freehand breaches medial cortical layer of a pedicle. (B) Mean cortical breach distances in the freehand and PPST groups. ***p <0.001.

Breaches of the cortical layer by the pedicles in the freehand and PPST groups were evaluated (Fig 5A). In the freehand group, the rates of an intrapedicular screw insertion with and without breaching the pedicle cortical layer were 51.9% (14/27) and 48.1% (13/27), respectively. In contrast, the rates of screws inserted with and without breaching the pedicle cortical layer in the PPST group were 22.2% (6/21) and 77.8% (21/27), respectively.

Compared with the freehand technique, PPST significantly increased the rate of intrapedicular screw usage (χ^2^ = 15.6; p <0.0001). The average wall penetration distance was significantly reduced by PPST compared with the freehand method (0.80 ± 0.17 mm in the freehand group *vs* 0.28 ± 0.12 mm in the PPST group; p = 0.0071), which indicated that pedicle wall cortex penetration could be significantly reduced by the PPST than the freehand technique (p <0.0071) (Fig 5B).

Screw trajectory was also evaluated using the frequently used Gertzbein and Robbins classification system [9] (Table 4). In the freehand group, the total rate of grade A (intrapedicular screw insertion without breaching the cortical layer of the pedicle; 13/27, 48.1%) and grade B (screw that breaches the pedicle cortical layer by ≤2 mm; 11/27, 40.7%) trajectories was 88.9%. Meanwhile, in the PPST group, the total rate of grades A and B cases was 96.3% (grade A: 21/27, 77.8%; grade B: 5/27, 18.5%). Grade C (penetration <4 mm) was observed in only one sample of the PPST group but was observed in three (11.1%) samples of the freehand group. No grade D or E cases were observed in either of the groups. All the grade C trajectories in both the freehand and PPST groups were in lateral direction positions. PS did not penetrate the rostral or caudal pedicle walls in either of the groups. Although PPST significantly reduced the distance of pedicle cortical breach, it did not significantly increase the acceptable rates defined by the Gertzbein and Robbins criteria compared to the freehand technique (χ^2^ = 1.08; p = 0.299).

**Table 4:** Screw positioning and direction of unacceptable wall penetration in the freehand and PPST groups according to the Gertzbein and Robbins classification system.

		Freehand n = 27	PPST n = 27
* **acceptable positioning**		24	26
** **unacceptable positioning**		3	1
**Wall penetration direction**	**Medial**	0	0
	**Lateral**	3	1
	**Rostral**	0	0
	**Caudal**	0	0

*Acceptable positioning corresponds to grades A and B based on the Gertzbein and Robbins classification system.

**unacceptable positioning corresponds to grades C, D, and E based on the Gertzbein and Robbins classification system.

## Discussion

We demonstrated that the accuracy of the PS trajectory and the safety of PS placement could be increased by following two steps: (1) having the axis of the target pedicle perpendicular to the ground and (2) performing pedicle probing and screwing perpendicular to the ground without any surgical support system. In the first step, the medial and rostral or caudal rotational angles of the longitudinal axes of the target pedicles should be measured in MPR mode on the DICOM viewer on an arbitrary cross-section. Such software costs less than 200 USD. Based on the measured rotational angle of the pedicle axis, rotation of the target pedicle by rotating the surgical table is necessary to position the pedicle axis perpendicular to the ground. This is feasible by attaching a three-axis accelerometer to a rotating surgical table, which can be purchased at less than 100 USD. The total cost for these items is extremely low than that of a CT-based navigation system.

Jost et al. and our group demonstrated a method for increasing the rotational accuracy of PS trajectories using a simple, low-cost support system by monitoring the attitude angles of surgical tools using an inertial measurement unit (IMU) sensor [7, 10]. However, this technique requires attaching the IMU indirectly to the pedicle probe, screwdriver, or jigs, thus requiring a sterilizable container to house the IMU to be prepared. Furthermore, the surgeon must check the IMU monitor outside the operating field to determine whether the rotational angles of the surgical tools and preoperatively planned rotational angles coincide, resulting in the dispersion of the surgeon’s eye points on different sites and consequently interrupting the surgical workflow.

Conversely, with PPST, positioning the target pedicle axis perpendicular to the ground is achieved by adjusting the rostrocaudal and mediolateral rotational angles of the surgical table by an individual who is neither the surgeon nor the assistant surgeon (e.g., OR nurse). Therefore, surgeons must focus only on maintaining the pedicle probe or screwdriver perpendicular to the ground. This means that the PPST can be performed more easily than the IMU-assisted method.

This study demonstrated that PPST helped reduce the offset between the preoperatively planned and postoperatively evaluated PS trajectories (Fig 4), resulting in reduced pedicle wall breach rates that can cause critical complications (Fig 5). Previous studies have demonstrated that the unacceptance rates of freehand PS placement in the thoracic and/or lumbar spine range from 9%–38% [11–16]. Although a simple comparison is inapplicable because our porcine spine samples were not the same as those in previous studies, the clinically unacceptable rate in our freehand groups was 11%, which is within the previously reported range. In comparison, the unacceptance rate in the PPST group was 4%, indicating the potential of PPST to reduce critical complications caused by PS placement.

Our study had several limitations. First, experimental data were obtained from the porcine lumbar spine. In PS research, the pig spine is a widely accepted model [17–20]; however, its morphological features do not completely match those of the human lumbar spine [21]. Therefore, the advantages of using PPST in human cadaver spines should be confirmed in the future. Second, because the patient’s body and surgical table pads have some elasticity in the actual clinical situation, micro-rotations and subsequent failure in positioning the target pedicle axis perpendicular to the ground may occur, especially when the rotational angle of the target pedicle axis is too large. To address this problem, the attitude angle of the patient’s back surface near the surgical field was monitored by attaching an IMU to check whether preoperatively planned anti-rotations were accurately performed. Although there are several issues to be solved before PPST can be clinically introduced for PS placement in human spine surgery, data from this study demonstrated that this simple technique may improve the safety and accuracy of PS placement.

## Conclusions

This study demonstrated the use of a simple PS placement technique based on the concept of an internal reference frame and Weber’s law of angle perception. The technique improved the accuracy and safety of PS placement in *ex vivo* porcine lumbar spines compared with the freehand method. Although the PPST technique needs to be further refined before it can be clinically introduced, it offers a potentially effective alternative to medical institutions that do not have access to expensive navigation systems for PS placement.
